# Are the Healthy Behaviors of US High-Deductible Health Plan Enrollees Driven by People Who Chose These Plans? Smoking as a Case Study

**DOI:** 10.1371/journal.pone.0056154

**Published:** 2013-02-13

**Authors:** Jeffrey T. Kullgren, Kevin G. Volpp, Daniel Polsky

**Affiliations:** 1 Veterans Affairs Center for Clinical Management Research, Veterans Affairs Ann Arbor Healthcare System, Ann Arbor, Michigan, United States of America; 2 Department of Internal Medicine, University of Michigan Medical School, Ann Arbor, Michigan, United States of America; 3 Institute for Healthcare Policy and Innovation, University of Michigan, Ann Arbor, Michigan, United States of America; 4 Leonard Davis Institute of Health Economics Center for Health Incentives and Behavioral Economics, Philadelphia, Pennsylvania, United States of America; 5 Penn-Carnegie Mellon University Roybal P30 Center on Behavioral Economics and Health, Philadelphia, Pennsylvania, United States of America; 6 Center for Health Equity Research & Promotion, Philadelphia Veterans Affairs Medical Center, Philadelphia, Pennsylvania, United States of America; 7 Department of Medicine, Perelman School of Medicine of the University of Pennsylvania, Philadelphia, Pennsylvania, United States of America; 8 Department of Health Care Management, the Wharton School, University of Pennsylvania, Philadelphia, Pennsylvania, United States of America; Erasmus University Rotterdam, The Netherlands

## Abstract

**Purpose:**

To determine whether negative associations between enrollment in a high-deductible health plan (HDHP) and one exemplar unhealthy behavior – daily smoking – are found only among people who chose these plans.

**Design:**

Cross-sectional analysis of nationally-representative data.

**Setting:**

United States from 2007 to 2008.

**Subjects:**

6,941 privately insured non-elderly adult participants in the 2007 Health Tracking Household Survey.

**Measures:**

Self-reported smoking status.

**Analysis:**

We classified subjects as HDHP or traditional health plan enrollees with employer-sponsored insurance (ESI) and no choice of plans, ESI with a choice of plans, or coverage through the non-group market. We used multivariate logistic regression to measure associations between HDHP enrollment and daily smoking within each of the 3 coverage source groups while controlling for potential confounders.

**Results:**

HDHP enrollment was associated with lower odds of smoking among individuals with ESI and a choice of plans (AOR 0.55, 95% CI 0.33–0.90) and those with non-group coverage (AOR 0.64, 95% CI 0.34–1.22), though the latter association was not statistically significant. HDHP enrollment was not associated with lower odds of smoking among individuals with ESI and no choice of plans (AOR 1.04, 95% CI 0.69–1.56).

**Conclusions:**

HDHP enrollment is associated with lower odds of smoking only among individuals who chose to enroll in an HDHP. Lower rates of unhealthy behaviors among HDHP enrollees may be a reflection of individuals who choose these plans.

## Introduction

One policy approach promoted as a way to reduce rates of unhealthy behaviors in the United States is greater enrollment in high-deductible health plans (HDHPs) [1], [2], which are private health insurance plans that feature deductibles of at least $1,100 per individual and $2,200 per family before most services are covered. Advocates for expansion of enrollment in HDHPs assert that placing patients at risk for the initial cost of their care through these plans encourages them to take greater responsibility for their health [3], [4].

The theoretical basis for believing HDHPs might change health behavior is based on the idea that because health insurance protects beneficiaries from facing the full financial consequences of medical care, beneficiaries might engage in more unhealthy behaviors than they would without this financial protection. This behavioral response to insurance, *ex ante* moral hazard, has little empirical support in the health services research literature [5], [6], [7], [8]. Nevertheless, the strong cross-sectional relationship between HDHP enrollment and lower rates of unhealthy behaviors [9], [10], [11], [12] has often been cited to support the belief that HDHPs leverage *ex ante* moral hazard to promote healthy behaviors.

An alternative explanation for these lower rates of unhealthy behaviors among HDHP enrollees is that individuals who engage in healthy behaviors at high rates choose HDHPs over traditional health insurance plans [13], [14], [15] because they expect to have few health expenditures and therefore are willing to accept high deductibles in exchange for the low monthly premiums characteristic of HDHPs [16]. If the lower odds of unhealthy behaviors among HDHP enrollees are driven largely by this individual plan self-selection instead of *ex ante* moral hazard, these lower odds would exist only among individuals who could choose their health plan and not among those who did not have a choice of health plans. However, it is currently unknown whether lower odds of any unhealthy behaviors are found just among HDHP enrollees who chose their plan. The objective of this study was to test whether choice of health plan, rather than *ex ante* moral hazard, can explain the healthier behaviors among HDHP enrollees by determining whether lower odds of one exemplar unhealthy behavior – daily smoking – are found only among HDHP enrollees who could choose their plan.

## Materials and Methods

### Ethics Statement

The study procedures were reviewed by the University of Pennsylvania Institutional Review Board (IRB) and deemed exempt from IRB review.

### Conceptual Framework

**Figure 1:**
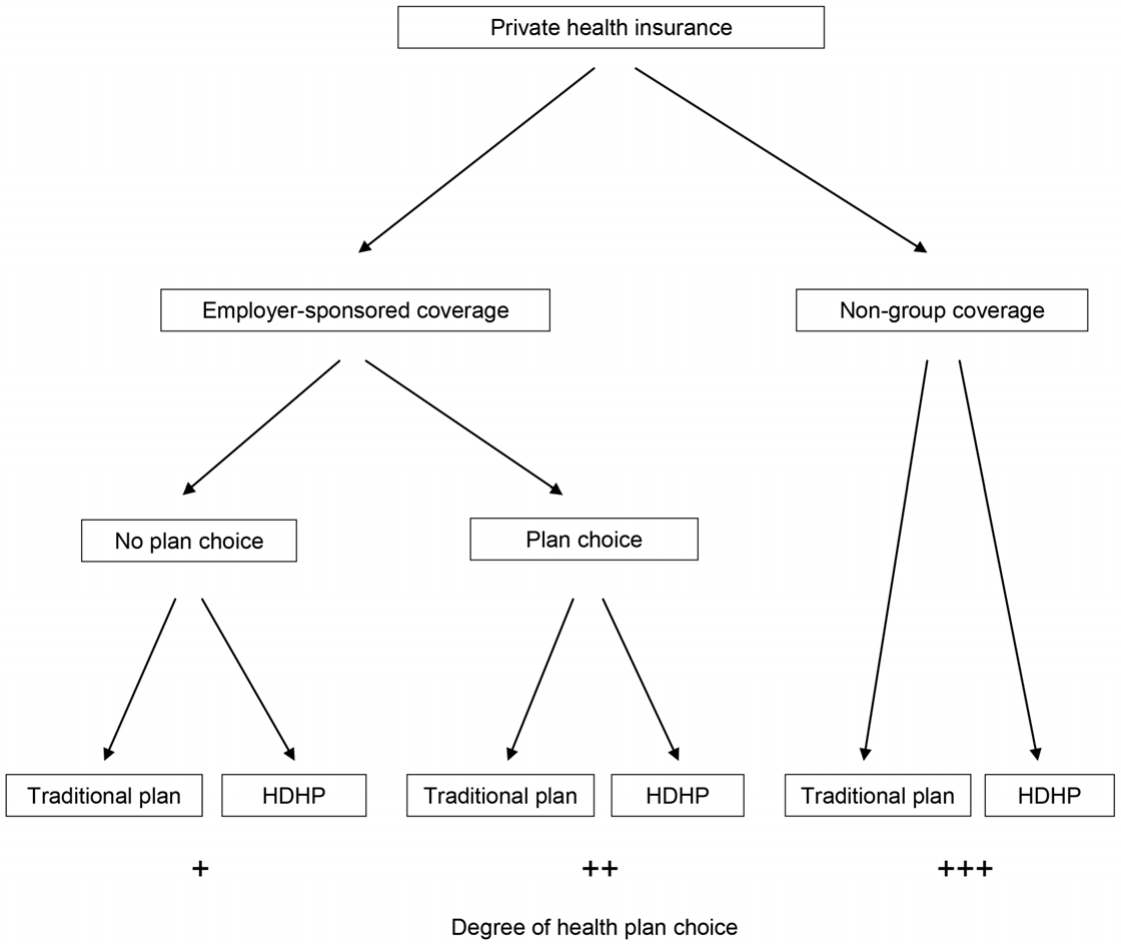
Conceptual framework. HDHP = high-deductible health plan.

Adults with pri**v**ate health insurance coverage in the United States have different degrees of health plan choice depending on whether they obtained their coverage from an employer who did not offer a choice of plans, an employer who offered a choice of plans, or the non-group market (Figure 1).

In the first group, employer-sponsored insurance (ESI) without plan choice, an individual is only offered one health insurance plan through the employer of an adult in their household. The employer chooses the plan to offer and the employee is left with a choice of opting in or not. Since there is no option to select among plans, this group contains the least potential to choose a health plan.

In the second group, employer-sponsored insurance (ESI) with plan choice, an individual has a choice between one or more health plans either because the employer of an adult in their household offers more than one plan to its employees or more than one adult in the household is eligible for health insurance through their employer. In this group, there is greater ability to choose a plan than in the ESI without plan choice group because an individual can select a plan from amongst several offerings [17], though the choice set is constrained by the range of plans offered to that household.

In the third group, non-group coverage, an individual purchases health insurance directly from an insurer. In this setting, there is the greatest potential to choose a plan, as an individual can select from amongst the many options the non-group market offers. The choice set is constrained only by medical underwriting [18] and an individual's willingness and ability to pay the quoted premiums.

Within each of these 3 coverage source groups, an individual is enrolled in either an HDHP or a traditional health insurance plan. The different degrees of health plan choice in each group offer a unique opportunity to explore the potential mechanisms underlying previously observed associations between HDHP enrollment and unhealthy behaviors such as smoking, as the choice to engage in an unhealthy behavior should not be influenced by an individual's degree of health plan choice. If, for example, the association between HDHP enrollment and an unhealthy behavior like smoking is driven primarily by *ex ante* moral hazard, then this association should be found in each coverage source group (i.e., irrespective of the degree of health plan choice). On the other hand, if the association between HDHP enrollment and an unhealthy behavior like smoking is driven more by selection of healthier individuals into HDHPs in settings of plan choice, then this association should be found only among individuals with the greatest ability to choose an HDHP (i.e., the ESI with plan choice and non-group coverage groups) and not among individuals with the least ability to choose an HDHP (i.e., the ESI without plan choice group).

### Data Source

The sample was comprised of 6,941 privately insured non-elderly adults in the 2007 Health Tracking Household Survey (HTHS). The 2007 HTHS was conducted between April 2007 and January 2008 by the Center for Studying Health System Change and Mathematica Policy Research, Inc., and used random digit dialing to collect data by telephone from 17,797 people in 9,407 households in the contiguous United States. The household response rate was 47.2 percent [19].

The survey collected demographic information and data on health insurance, employment, and health characteristics for each adult in every sampled household. If any individuals in the household had private health insurance, information was collected on who was covered by the plan, how that plan was obtained (i.e., through an employer or through purchase in the non-group market), and whether the plan had a deductible. If the plan had a deductible, information was collected on the size of the deductible. Individuals with employer-sponsored insurance (ESI) were asked whether that employer offered 1 health insurance plan to its employees or more than 1 plan; no data were collected on the types of plans the employer offered to that individual. Those who were employed but not a policyholder of an employer-sponsored plan were asked whether they were eligible for health insurance through their employer. Each adult respondent was also asked about his or her smoking; those who indicated they had smoked at least 100 cigarettes in their entire life were asked whether they currently smoke cigarettes every day, some days, or not at all.

### Main Predictor Variables

For analysis, we divided individuals into 1 of 3 coverage source groups discussed in the Conceptual Framework section.

The first group was ESI without plan choice. We assigned individuals to this group if they were enrolled in an ESI plan through an employer that did not offer a choice of health plans and no one else in that family insurance unit [19] was eligible for employer-sponsored coverage.

The second coverage source group was ESI with plan choice. We assigned individuals to this group if they had ESI and either the employer from which the coverage was obtained offered a choice of plans or another adult in the family insurance unit was also eligible for coverage through their employer.

The third coverage source group was individuals with non-group coverage. We assigned individuals to this group if they had private health insurance coverage that was not obtained through an employer.

In each of these 3 coverage source groups, we classified individuals as being enrolled in an HDHP as defined by US federal law: a private health insurance plan with an annual individual deductible of at least $1,100 or a family deductible for at least $2,200 [20]. All other privately-insured individuals in the sample were classified as traditional plan enrollees.

### Primary Outcome Variable

The primary outcome variable was specified *a priori*. Individuals who stated they currently smoke cigarettes every day were classified as daily smokers.

### Covariates

Data on gender, age, annual household income, race/ethnicity, education, chronic conditions, time in the current health insurance plan, risk-taking, employment status, marital status, parental status, and US Census region were obtained from the survey. County metropolitan statistical area category was obtained from the 2007 Area Resource File. All covariates were operationalized as categorical variables with mutually-exclusive categories. Age was defined as 3 categories: 18 to 25 years, 26 to 45 years, or 46 to 64 years. Annual household income was operationalized as 3 categories: less than $50,000; $50,000 to $100,000; or greater than $100,000. Race and ethnicity data were collected in categories used in the US Census. Education was operationalized as a dichotomous variable based on whether an individual had at least 16 years of education (i.e., a college degree). Having a chronic condition was operationalized as a dichotomous variable based on whether an individual reported a history of heart disease, cancer, diabetes, chronic obstructive pulmonary disease, hypertension, arthritis, asthma, or depression. Length of time in one's current health insurance plan was defined as more than 12 months or less than 12 months. Respondents who agreed with the statement, “I'm more likely to take risks than the average person” were classified as risk-takers. Employment status was operationalized as 3 categories: full-time, part-time, or not working.

### Statistical Analysis

We first tested for differences in the characteristics and smoking rates of HDHP and traditional health plan enrollees using Pearson chi square tests. We then estimated the odds ratios for the key relationships of interest using logistic regression models to predict daily smoking. We first measured the overall unadjusted association between HDHP enrollment and daily smoking using a univariate model. We then examined whether the association between HDHP enrollment and daily smoking differed by the degree to which individuals could choose a plan by estimating the interaction of HDHP enrollment and coverage source. In this multivariate model we included the aforementioned *a priori* set of covariates to control for previously identified observable differences between individuals in each coverage source group [21], [22], [23] as well as correlates of smoking [24], [25], [26], [27] and modifying factors in the Health Belief Model [28] that could potentially confound the relationship between plan type and daily smoking. Finally, we performed post-estimation linear contrasts from the multivariate model to compare associations between HDHP enrollment and daily smoking within each of the 3 coverage source groups (i.e., among individuals with the same ability to choose a plan).

Several approaches were taken to address missing data. The 1,155 HTHS respondents who were privately insured non-elderly adults but had missing data for smoking status were excluded from the analyses. Missing values for ESI offers and eligibility, household income, race/ethnicity, education, and employment status were previously imputed by the Center for Studying Health System Change and Mathematica Policy Research, Inc. using unweighted and weighted sequential hot-deck imputation [19]. Between 0.01 percent and 3.8 percent of the values for these covariates were imputed, with the exception of household income, for which 23.6 percent of the values were imputed.

We used Stata 11 (StataCorp. 2009. *Stata Statistical Software: Release 11*. College Station, TX: StataCorp LP) for all analyses. Nationally-representative estimates were constructed by applying sample weights that account for the sampling design and survey non-response [19], [29]. In all cases we used a pre-specified α = 0.05 to indicate statistical significance.

## Results

### Sample Characteristics

Table 1 shows the characteristics of HDHP enrollees (n = 1,111) and traditional plan enrollees (n = 5,830). HDHP enrollees were less likely than traditional plan enrollees to be smokers (9.1% vs. 12.2%, *P* = 0.02). Enrollees in HDHPs were also less likely than traditional plan enrollees to be African American (5.1% vs. 9.5%, *P* = 0.001), living in a Metropolitan area (84.1% vs. 87.0%, *P* = 0.04), and living in the Northeast region (11.4% vs. 19.7%, *P*<0.001); and more likely than traditional plan enrollees to be White (81.4% vs. 76.3%, *P* = 0.02), not working (28.3% vs. 24.2%, *P* = 0.008), parents (58.3% vs. 51.9%, *P* = 0.006), and living in the Midwest region (30.9% vs. 25.0%, *P* = 0.004).

**Table 1:** Characteristics of privately insured US adults by plan type, 2007–2008*.

	Traditional plan	HDHP	*P*-value
N	5,830	1,111	
Weighted N	82,317,300	14,411,916	
Female, %	53.3	52.1	0.38
Age, %			
18–25 years old	13.2	12.8	0.76
26–45 years old	42.5	41.8	0.77
46–64 years old	44.3	45.4	0.58
Annual household income, %			
<$50,000	27.6	25.0	0.20
$50,000 to $100,000	39.9	40.5	0.80
>$100,000	28.4	29.6	0.59
Race/ethnicity, %			
White	76.3	81.4	0.02
African-American	9.5	5.1	0.001
Hispanic	8.8	7.4	0.33
Other non-Hispanic	5.4	6.1	0.56
College education, %†	36.6	40.1	0.08
Employment status, %			
Full time	57.7	55.2	0.13
Part time	18.1	16.5	0.22
Not working	24.2	28.3	0.008
Married, %	75.2	76.6	0.45
Parent, %	51.9	58.3	0.006
Census region, %			
Northeast	19.7	11.4	<0.001
Midwest	25.0	30.9	0.004
South	33.7	34.6	0.71
West	21.6	23.1	0.47
Metropolitan statistical area category, %			
Metropolitan	87.0	84.1	0.04
Micropolitan	8.2	9.8	0.14
Non-statistical area	4.8	6.1	0.18
Fair or poor health status, %	9.8	9.8	0.97
Chronic condition, %‡	45.9	43.5	0.23
Daily smoker, %§	12.2	9.1	0.02

HDHP = high-deductible health plan.

* Unless otherwise noted, data are weighted proportions.

† At least 16 years of education.

‡ Diabetes, arthritis, asthma, chronic obstructive pulmonary disease, hypertension, heart disease, cancer other than skin cancer, or depression.

§ Currently smoking cigarettes every day.

**Figure 2:**
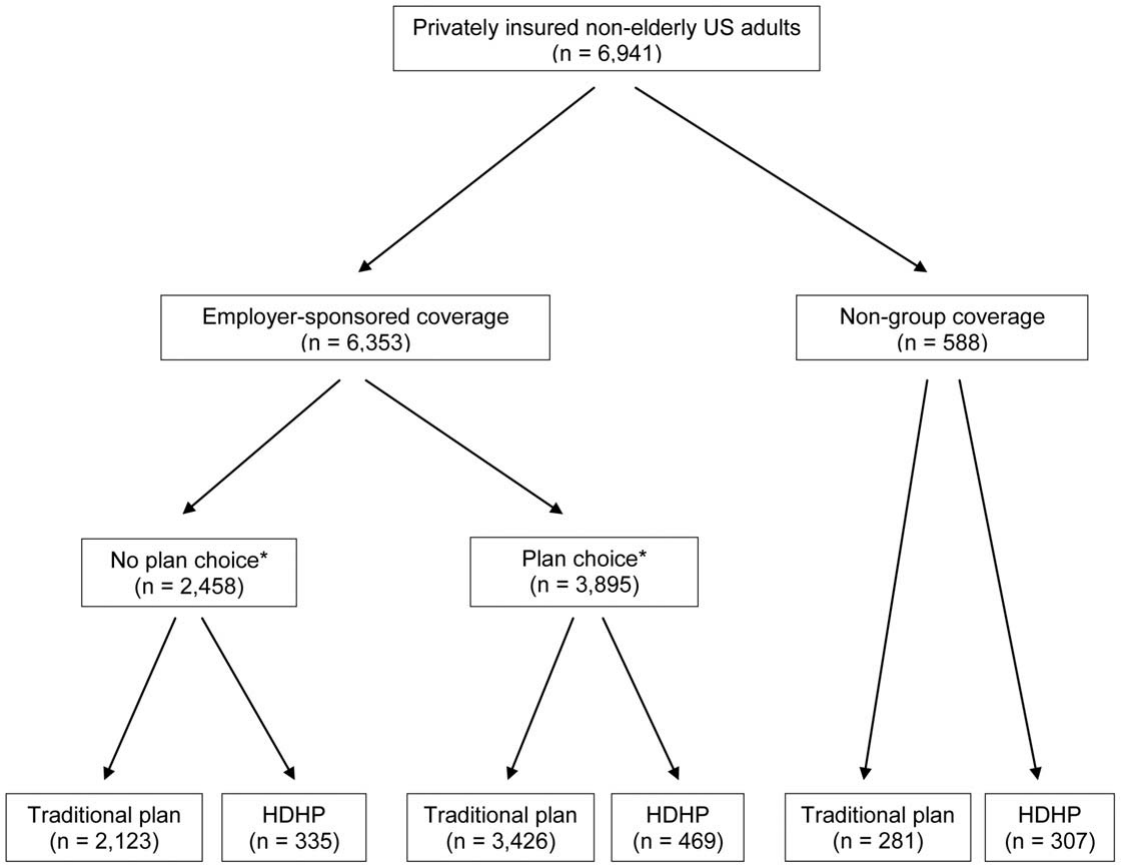
Study sample. HDHP = high-deductible health plan. * Plan choice defined as the plan policyholder had a choice of health insurance plans through his or her employer and/or a non-policyholder in the family insurance unit was also eligible for coverage through his or her employer.

Figure 2 shows the coverage sources and counts for HDHP and traditional plan enrollees in the analytic sample. Among the 2,458 individuals who had ESI without plan choice, 13.6% were enrolled in an HDHP. Among the 3,895 who had ESI with plan choice, 12.0% were in an HDHP. Among the 673 who had non-group coverage, 52.2% were enrolled in an HDHP.

### Associations between Plan Type and Daily Smoking by Coverage Source

**Figure 3:**
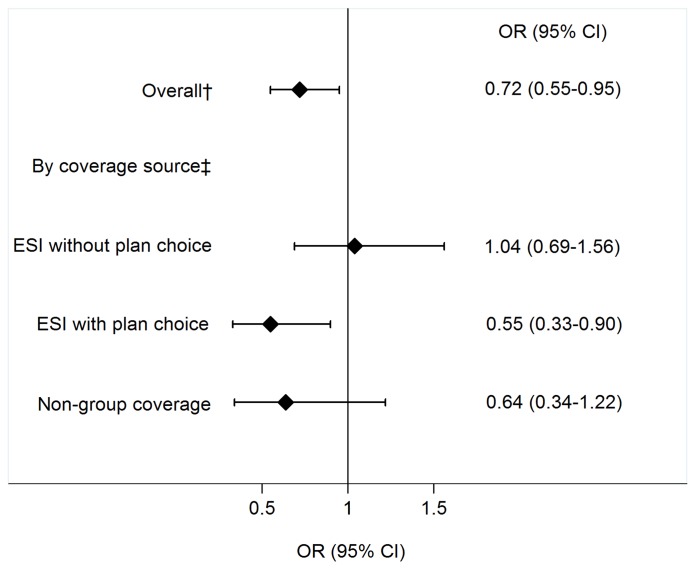
Odds of smoking* for HDHP vs. traditional plan enrollees: overall and by coverage source. HDHP = high-deductible health plan. ESI = employer-sponsored insurance. * Smoking defined as currently smoking cigarettes every day. † Unadjusted odds of being a daily smoker among all HDHP enrollees. Reference group is all traditional plan enrollees. ‡ Odds of being a daily smoker adjusted for gender, age, household income, race/ethnicity, chronic conditions, time in current health insurance plan, risk tolerance, employment status, marital status, parental status, US Census region, and metropolitan statistical area category. Reference group is traditional plan enrollees in the same coverage source group.

Figure 3 shows the main results from the 2 logistic regression models. In the univariate model, HDHP enrollment was associated with lower overall odds of being a daily smoker (odds ratio 0.72, 95% CI 0.55–0.95). In the multivariate model, HDHP enrollment was associated with lower odds of being a daily smoker among individuals with ESI and plan choice [adjusted odds ratio (AOR) 0.55, 95% CI 0.33–0.90] and individuals with non-group coverage (AOR 0.64, 95% CI 0.34–1.22), though the latter association was not statistically significant. HDHP enrollment was not associated with lower odds of being a daily smoker among individuals with ESI and no plan choice (AOR 1.04, 95% CI 0.69–1.56).

## Discussion

HDHP enrollment is associated with lower overall odds of being a daily smoker. However, these lower odds of daily smoking exist only among individuals who chose their health plan, and not among those who could not choose their health plan. To our knowledge this is the first study to explore whether lower odds of any unhealthy behavior are found only among HDHP enrollees who chose that type of plan.

HDHPs have been promoted as a policy tool for reducing modifiable behavioral risk factors like smoking and obesity [1], [2], [3], [4], and previous analyses have shown associations between enrollment in these plans and lower rates of unhealthy behaviors [9], [10], [11], [12]. When evaluating the potential for policy tools to effectively reduce behavioral risk factors, however, it is essential to consider other factors that could explain observed associations between the intervention and the desired outcome [15]. In the case of associations between HDHP enrollment and behavioral risk factors like smoking, the role of individual plan self-selection is potentially very important. Many privately insured individuals have a choice of health plans, and those who already engage in healthy behaviors (e.g., being a non-smoker, eating a healthy diet, or being physically active) may be particularly willing to accept the higher financial risk of HDHPs in return for lower monthly premiums.

Our results provide stronger evidence for this plan selection effect than for a health-promoting effect of HDHPs, since HDHP enrollment was associated with lower odds of daily smoking only among individuals who chose this type of plan. We have no reason to believe there are differences in the exposures or measurement of the outcomes that could explain the differences in associations we observed across the 3 coverage source groups. Hence, after multivariate adjustment for differences between individuals in each group, the principal remaining difference across these groups is the potential to self-select into a plan.

These results carry important implications for policymakers and employers who are increasingly searching for new tools like insurance benefit design to reduce rates of unhealthy behaviors [16], [30]. Our findings suggest that offering only an HDHP to employees with the expectation that this will reduce rates of unhealthy behaviors like smoking – as a growing number of employers are now doing [31], [32] – may not have its intended effects. These results are also consistent with findings from the RAND Health Insurance Experiment, where there were no lower rates of unhealthy behaviors observed among individuals who had been randomly assigned to plans with high levels of cost-sharing [33].

This study has limitations. First, our conceptual framework assumes that individuals enter the three groups (ESI without plan choice, ESI with plan choice, and non-group coverage) randomly. If group selection, even indirectly, depends on smoking behavior, potential unmeasured confounders limit the ability to infer causality. The fact that our results are not sensitive to measured potential confounders, however, leads us to believe that this is not a serious limitation. Second, the smaller sample size of the 2007 HTHS as compared with previous rounds of this survey limits the precision of parameter estimates for certain sub-groups, such as individuals with non-group coverage. The 2007 HTHS, however, is the only nationally-representative survey to date that has concurrently collected data on an individual's health plan deductible, degree of plan choice, and any health behavior. Third, while we were able to classify privately insured adults into 3 groups based on their degree of plan choice, we were not able to observe the actual composition of each individual's plan choices or their reasons for choosing their particular plan. Finally, these are self-reported data that are subject to recall bias. However, there is no clear reason why recall bias would differentially affect people in relation to their plan type or coverage source.

This study adds new data on the relationship between HDHP enrollment and unhealthy behaviors among US adults. While HDHP enrollment is associated with lower overall odds of daily smoking, these lower odds exist only among individuals who chose their plan. Therefore, lower rates of unhealthy behaviors such as smoking among HDHP enrollees may be a reflection of individuals who choose these plans.
